# Impact of intraoperative haemoadsorption on outcomes of patients undergoing aortic surgery: a single-centre, prospective, observational study

**DOI:** 10.1093/icvts/ivae050

**Published:** 2024-03-21

**Authors:** Yatin Mehta, Ajmer Singh, Mandeep Singh, Anil Bhan, Naresh Trehan

**Affiliations:** Institute of Critical Care and Anesthesiology, Medanta—The Medicity, Gurugram, India; Institute of Critical Care and Anesthesiology, Medanta—The Medicity, Gurugram, India; Institute of Critical Care and Anesthesiology, Medanta—The Medicity, Gurugram, India; Department of Cardiac Surgery, Medanta—The Medicity, Gurugram, India; Department of Cardiac Surgery, Medanta—The Medicity, Gurugram, India

**Keywords:** Aortic surgery, Haemoadsorption, Cytokine removal, CytoSorb^®^

## Abstract

**OBJECTIVES:**

To investigate the impact of a cytokine haemoadsorption (HA) device (CytoSorb^®^) on inflammatory markers and patients’ outcome during aortic root surgery.

**METHODS:**

Prospective, observational study including all-comers with quasi-randomization by strictly alternating inclusion (1:1 basis). Sixty patients undergoing elective aortic surgery were assigned to either HA group (*n* = 30) with intraoperative HA, or a control (C) group (*n* = 30). Primary outcomes were: (i) impact of HA on haemodynamic stability and need for vasopressors (vasoactive–inotropic score) and (ii) sequential organ failure assessment (SOFA) score. Secondary parameters included the impact of HA on the course of hyperinflammation using interleukin-6 and procalcitonin, duration of mechanical ventilation, and lengths of intensive care unit and hospital stay.

**RESULTS:**

Noradrenaline requirement was significantly reduced in the HA group postoperatively compared to the C group (HA: 0.03 µg/kg/min vs C: 0.08 µg/kg/min, *P* = 0.004 at 2 h, and HA: 0.02 µg/kg/min vs C: 0.04 µg/kg/min, *P* = 0.004 at 24 h). This translated into a significantly lower vasoactive–inotropic score in the HA group. SOFA score was less in the HA group at all time points and reached statistical significance 2 h postoperatively (HA: 5.77 vs C: 7.43, *P* < 0.001). Intraoperative HA significantly reduced interleukin-6 levels (*P* < 0.05) at all time points, and procalcitonin at 2 h after discontinuation from cardiopulmonary bypass (*P* = 0.005). The duration of ventilation, intensive care unit and hospital stays were shorter in the HA group compared to the C group.

**CONCLUSIONS:**

Intraoperative HA has the potential to mitigate hyperinflammatory response leading to improved haemodynamics after aortic root surgery, thereby shortening the duration of ventilation, and lengths of intensive care unit and hospital stay. However, it must be evaluated in larger cohorts.

**Figure:**
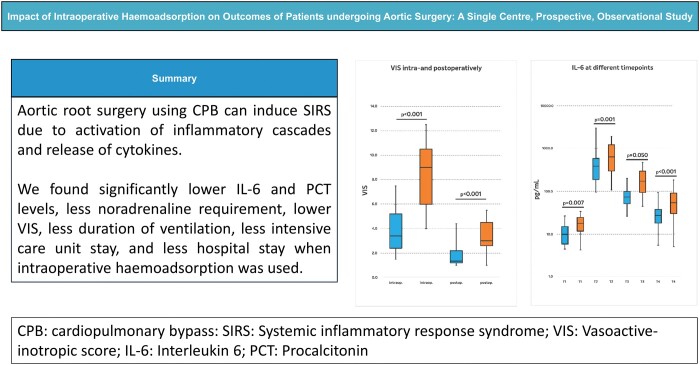

## INTRODUCTION

Cardio-surgical procedures using cardiopulmonary bypass (CPB) can induce a systemic inflammatory response syndrome due to activation of inflammatory cascades. Profound surgical trauma induced by major surgery such as cardiac surgery, along with bacterial spread-out, and artificial CPB surfaces leads to activation of both humoral and cellular immune responses [1]. Activation of various mediators of inflammation such as interleukin (IL) 1-β, IL-4, IL-6, IL-8, IL-10 and tumour necrosis factor-alpha can lead to postoperative complications. In severe cases, this can lead to vasoplegic shock characterized by an increased cardiac output (CO) and decreased systemic vascular resistance, requiring treatment with vasopressors and intravenous fluids. These complications can cause organ dysfunction, delayed weaning from mechanical ventilation and prolonged intensive care unit (ICU) stay [2]. Thus, measures to decrease the inflammatory process have the potential to improve perioperative outcome with reduced morbidity and mortality.

Haemoadsorption (HA) using the CytoSorb^®^ adsorber is a recent, promising technology that has shown rapid elimination of many key cytokines, modulation of inflammatory response and improved outcome [3–5]. Removal of inflammatory mediators has the potential to attenuate the deleterious effects of the dysregulated inflammatory response while preserving the ability of the patient’s immune system to mount an appropriate defence against these challenges during the perioperative period [6].

The aim of the present study was, therefore, to evaluate the effect of intraoperative HA in patients undergoing major aortic root surgery.

## MATERIAL AND METHODS

### Ethics statement

The study was approved by the institutional ethics committee (Medanta Institutional Ethics Committee). The approval number was 1424/2022 (DNB), 19 August 2022 and the trial was registered at CTRI/2022/11/047314. (https://ctri.nic.in/Clinicaltrials/showallp.php?mid1=76159&EncHid=&userName=047314). Written informed consent was obtained from each patient.

### Study design

We prospectively investigated 60 consecutive adult patients undergoing elective surgery for ascending aortic aneurysm including aortic root surgery. This observational study was conducted at a tertiary care medical centre. Sample size calculation was performed based on the results of our previous pilot study [5]. The minimum sample size of 95% confidence interval corresponding to an 80% power resulted in 28 patients per group, which was increased (safety margin) to 30 patients per group. Inclusion criteria were: (i) consecutive adult patients (all-comers) with ascending aortic aneurysm admitted for elective aortic root surgery with (ii) an expected CPB duration of more than 120 min. Exclusion criteria were: (i) emergency/urgent procedures, (ii) aortic dissection, (iii) infective endocarditis, (iv) history of stroke, (v) scheduled insertion of a cardiac assist device, (vi) patients receiving chemotherapy/immunosuppressants/steroids or anti-leucocyte drugs or (vii) moribund patients.

In the study group (HA group), a CytoSorb adsorber (CytoSorbents Inc., Princeton, NJ, USA) was used for the entire duration of CPB. The results obtained from these patients were compared to a similar number of patients (C group) in whom intraoperative HA was not applied. Patients were included by quasi-randomization with strictly alternating treatment (1:1 ratio).

Using a standard pre-anaesthetic regimen, invasive haemodynamic monitoring including a pulmonary artery catheter, and standard anaesthetic technique, all surgical procedures were performed under hypothermic CPB. CPB management consisted of crystalloid primed, non-pulsatile flow (2.4 l/min/m^2^), non-heparin-coated circuit, membrane oxygenator and moderate hypothermia (28–30°C). After arterial cannulation (right axillary artery or distal ascending aorta, depending on the space available) venous cannulation, institution of CPB and delivery of cardioplegia, the proximal aortic reconstruction (root replacement) was performed 1st. The distal anastomosis was completed using either the same graft as used for the proximal or a separate graft sewn to the proximal graft taking care that the graft(s) do not kink. All patients were monitored with cerebral oximetry throughout the surgery. In the HA group, a 300 ml CytoSorb adsorber was installed between the venous reservoir and the oxygenator, and blood was pumped actively through the cartridge by using a centrifugal pump. A roller pump ensured a standardized flow of 400 ml/min through the cartridge in all treated patients. In the control group, no adsorber was used.

Blood samples were collected for IL-6 levels, procalcitonin (PCT), total leucocyte count, lactate and serum creatinine at 4 pre-defined time points. The methodology used for measuring ILs was direct enzyme-linked immunosorbent assay, and for PCT, it was enzyme-linked fluorescence assay. Timepoint definition was: T_1_: baseline, after induction of anaesthesia, T_2_: 2 h after discontinuation of CPB, T_3_: 24 h after discontinuation of CPB and T_4_: 48 h after discontinuation of CPB. Haemodynamic parameters and requirement for vasopressors were also assessed at these time points.

### Endpoint definition

The primary outcomes were: (i) impact of HA on haemodynamic stability and need for vasopressors (vasoactive–inotropic score: VIS) and (ii) the sequential organ failure assessment (SOFA) score. Secondary parameters were IL-6 levels, PCT levels, leucocyte count, lactate, mean arterial pressure (MAP), CO, systemic vascular resistance, PaO_2_:FiO_2_ ratio (PF ratio), duration of mechanical ventilation, length of stay in the ICU and hospital.

The VIS was calculated using the method described by Gaies *et al.* [7]. The MAP was kept >65 mmHg by use of intravenous fluids and vasopressors. The decision to discontinue from mechanical ventilation was taken by the ICU team. All patients were extubated to venturi face mask ventilation. The decision to discharge from the ICU and hospital was taken by the operating surgeon.

### Statistics

Data were analysed using the SPSS software package, version 26 (SPSS Inc., Chicago, IL, USA). Data were summarized as mean and standard deviation or median and interquartile range as indicated by sample size and/or data distribution. Discrete and categorical data were reported as frequencies and percentages. Differences between groups were analysed by using the Student’s *t*-test for continuous variables and Fisher’s exact test for categorical variables. A *P*-value of ≤0.05 was considered to be statistically significant.

## RESULTS

Between August 2022 and August 2023, a total of 60 patients were enrolled in the study, 30 in the HA group and 30 in the C group. There were no dropouts. The patient characteristics, including EuroSCORE-II, ejection fraction and New York Heart Association class, were well matched in both groups (Table 1), except for body mass index. Patients were predominantly male in both groups and the majority had ascending aortic aneurysms with severe aortic regurgitation. The remaining patients had ascending aortic aneurysms with aortic stenosis, concomitant mitral stenosis or regurgitation, or coronary artery disease. All patients underwent a Bentall-de Bono procedure, either isolated or as a combined procedure for the associated lesion, by a single surgeon (Table 2). Since none of the patients required hemiarch reconstruction, hypothermic circulatory arrest or isolated cerebral perfusion were not required. Four patients in the HA group and 2 patients in the C group underwent redo cardiac surgery. Associated comorbid conditions are shown in Table 3. One patient with COVID-19 infection required surgery for aortic root dehiscence. The surgery was performed on a priority and we did not wait for the patient to fully recover from COVID-19 illness as this could have worsened his cardiac condition.

**Table 1:** Patients characteristics (mean with standard deviation or numbers with percentage)

Parameter	Haemoadsorption group (*n*=30), mean ± SD	Control group (*n*=30), mean ± SD	Mean ± SE of difference	95% CI of the difference (lower–upper)	*P*-value
Age (years)	48.23 (SD: 14.87)	49.5 (SD: 12.26)	–1.27 ± 3.52	–8.31 to 5.78	0.720
Male: Female	26:4 (86.7% vs 13.3%)	26: 4 (86.7% vs. 13.3%)	–	–	1.000
BMI (kg/m^2^)	24.47 (SD: 3.42)	27.4 (SD: 4.87)	–2.93 ± 1.09	–5.11 to –0.75	0.009
BSA (m^2^)	1.81 (SD: 0.23)	1.92 (SD: 0.21)	–0.11 ± 0.06	–0.22 to 0.0	0.055
EuroSCORE-II	2.9 (SD: 2.85)	2.79 (SD: 4.62)	–1.87 ± 2.1	–1.87 to 2.1	0.911
Ejection fraction (%)	49.5 (SD: 8.65)	49.2 (SD: 7.98)	0.3 ± 2.15	–4.0 to 4.6	0.889
NYHA class					
II	13 (43%)	16 (53.3%)	–	–	0.850
III	16 (53.3%)	14 (46.7%)	–	–	0.895
IV	1 (3.3%)	0 (0%)	–	–	0.500

BMI: body mass index; BSA: body surface area; CI: confidence interval; EuroSCORE-II: European system for cardiac operative risk evaluation II; NYHA: New York Heart Association; SD: standard deviation; SE: standard error.

**Table 2:** Preoperative diagnosis and surgical procedures (number of patients with percentage)

Parameter	HA group (*n* = 30)	Control group (*n* = 30)	*P* value
Preoperative diagnosis			
AA aneurysm, severe AR	15 (50%)	12 (40%)	0.785
AA aneurysm, severe AS	2 (6.66%)	3 (10%)	0.669
AA aneurysm, BAV	2 (6.66%)	5 (16.6%)	0.386
AA aneurysm, Marfan syndrome	4 (13.3%)	5 (16.6%)	0.733
AA aneurysm, CAD	1 (3.33%)	1 (3.33%)	0.981
AA aneurysm, MS	1 (3.33%)	0 (0%)	0.500
AA aneurysm, MR	1 (3.33%)	2 (6.66%)	0.554
Post Bentall root dehiscence	1 (3.33%)	0 (0.0%)	0.500
AA aneurysm, post AVR	2 (6.66%)	2 (6.66%)	1.000
AA aneurysm, Post DTA aneurysm	1 (3.33%)	0 (0.0%)	0.500
Surgical procedures			
Bentall procedure	23 (76.6%)	25 (83.3%)	0.685
Redo Bentall	4 (13.3%)	2 (6.6%)	0.315
Bentall + MVR	2 (6.6%)	2 (6.6%)	1.000
Bentall + CABG	1 (3.3%)	1 (3.3%)	1.000
**Mortality**	0 (0%)	0 (0%)	–

AA: ascending aortic; AR: aortic regurgitation; AS: aortic stenosis; AVR: aortic valve replacement; BAV: bicuspid aortic valve; CABG: coronary artery bypass grafting; CAD: coronary artery disease; DTA: descending thoracic aorta; HA: haemoadsorption; MR: mitral regurgitation; MS: mitral stenosis; MVR: mitral valve replacement.

**Table 3:** Associated comorbid conditions (numbers and percentage)

Co-morbid conditions	Haemoadsorption group (*n* = 30)	Control group (*n* = 30)	Total (*n* = 60)	*P*-value
Hypertension	15 (50%)	14 (46.6%)	29 (49.3%)	0.895
Diabetes mellitus	7 (23.3%)	6 (20%)	13 (21.6%)	0.785
Hypothyroid	3 (10%)	4 (13.3%)	7 (11.6%)	0.652
COPD/smoker	5 (16.6%)	5 (16.6%)	10 (16.6%)	0.959
COVID-19				
Previous	4 (13.3%)	5 (16.6%)	9 (15%)	0.733
Current	1 (3.3%)	0 (0%)	1 (1.6%)	0.156
Renal insufficiency	6 (20%)	3 (10%)	9 (15%)	0.302
Preop RRT	2 (6.7%)	2 (6.7%)	4 (6.7%)	1.000
Previous cardiac surgery	4 (13.3%)	2 (6.7%)	6 (20%)	0.315

COPD: chronic obstructive pulmonary disease; COVID-19: corona virus disease-19; RRT: renal replacement therapy.

**Figure 1:**
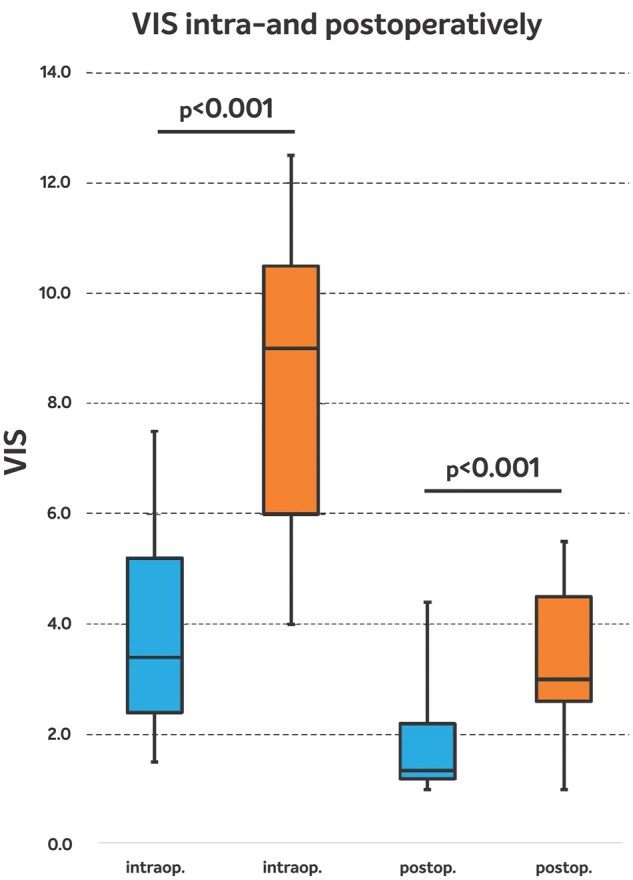
Vasoactive–inotropic score (VIS). intra-op: intraoperatively at 2 h after cardiopulmonary bypass (T_2_); post-op: postoperatively at 24 h after cardiopulmonary bypass (T_3_), HA: blue, C: orange.

The VIS (Fig. 1) and noradrenaline requirement (Table 4) measured at T_2_ (intraoperative) and T_3_ (postoperative) time points were significantly less in the HA group compared to the controls (*P* < 0.001 and *P* = 0.004, respectively). The SOFA score was less in the HA group at all time points and reached statistical significance at T_2_ (HA: 5.77 vs C: 7.43, *P* < 0.001).

**Table 4:** Median values with interquartile range (IQR) for procalcitonin, lactate levels and noradrenaline requirement (µg/kg/min)

Parameter	HA group (*n* = 30), median (IQR)	Control group (*n* = 30), median (IQR)	*P*-value
Procalcitonin (ng/ml)			
T_1_	0.05 (0.05–0.13)	0.05 (0.05–0.54)	0.732
T_2_	5.62 (2.33–9.74)	11.93 (5.02–25.1)	0.005
T_3_	6.45 (4.24–9.86)	7.66 (3–13.4)	0.539
T_4_	4.31 (2.55–7.07)	2.96 (1.27–6.43)	0.237
Lactate (mmol/L)			
T_1_	1.54 (0.91–2.39)	1.51 (0.9–2.17)	0.910
T_2_	4.09 (3.77–5.22)	5.06 (2.91–7.33)	0.322
T_3_	2.32 (1.61–3.06)	2.96 (1.49–4.3)	0.234
T_4_	1.2 (0.98–1.56)	1.46 (0.9–2.12)	0.604
Noradrenaline requirement			
Intraoperative (T_2_)	0.03 (0.02–0.05)	0.08 (0.03–0.17)	**0.004**
Postoperative (T_3_)	0.02 (0.01–0.03)	0.04 (0.02–0.07)	**0.004**

T_1_: post-induction, T_2, 3, 4_: at 2, 24, 48 h after cardiopulmonary bypass; HA: Haemoadsorption.

Bold/italic values represents statistically significant p value.

**Figure 2:**
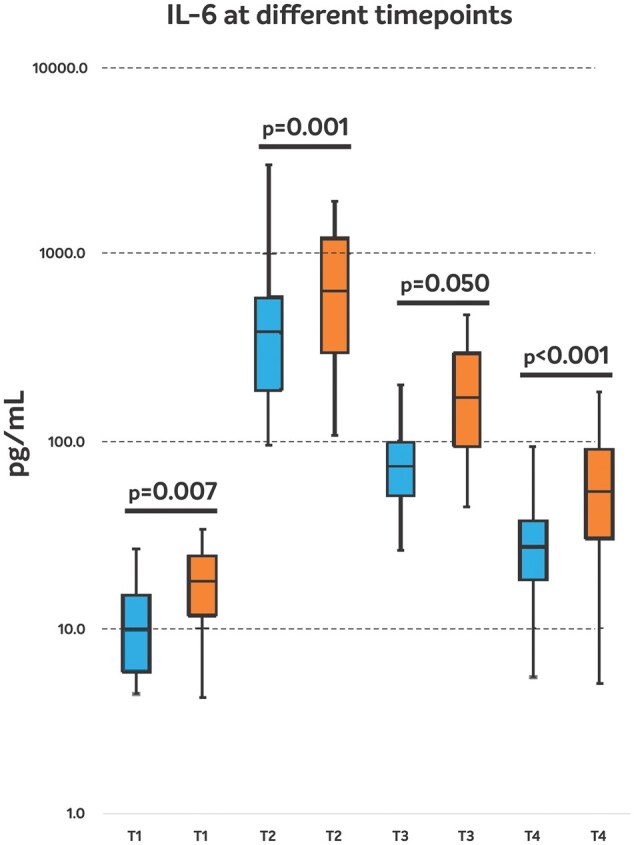
Median values of interleukin-6 (pg/ml). IL-6: interleukin-6 in pg/mL on a logarithmic scale, HA: blue, C: orange, T_1_: post-induction, T_2, 3, 4_: at 2, 24, 48 h after cardiopulmonary bypass.

The course of IL-6 levels is presented in Fig. 2. Levels of IL-6 were significantly lower in the HA group compared to the C group at all time points. Compared to the baseline values (T_1_), there was an increase in IL-6 levels in both groups, with peak values observed at T_2_ but levels were significantly lower in the HA group as compared to controls (HA: 383.85 pg/ml vs C: 638.35 pg/ml, *P* = 0.001). The IL-6 levels remained significantly lower in the HA group compared to the C group at T_3_ and T_4_ (*P* = 0.050 and *P* < 0.001, respectively). PCT levels were similar in the 2 groups at T_1_, but the levels were significantly less at T_2_ in the HA group (HA: 5.62 ng/ml vs C: 11.93 ng/ml, *P* = 0.005). Lactate levels showed lower values in the HA group compared to C group at T_2–4_ time points. However, these differences did not reach statistical significance. Total leucocyte count (not shown in the table) did not show any difference between the groups. PCT and lactate levels are given in Table 4.

CPB duration and aortic cross-clamp time were similar in both groups (Table 5). The duration of mechanical ventilation was shorter in the HA group compared to the control group (HA: 13.63 h vs C: 20.03 h, *P* < 0.001). Similarly, the length of stay in the ICU and the hospital were significantly shorter in the HA group compared to C group (*P* = 0.006 and *P* = 0.001, respectively). The PF ratio was significantly higher in the HA group compared to C group at T_2–4_ (*P* < 0.001, Table 6). The haemodynamic parameters (MAP, central venous pressure, CO, CI, systemic vascular resistance) did not show any clinically important difference, except for the finding that the HA group patients were little more vasoconstricted at T_1_ (Table 7). There were no HA device-related adverse events, and at a 30-day follow-up, there was no mortality in either group.

**Table 5:** Comparison of perioperative variables between the groups

Variable	Haemoadsorption group (*n* = 30), mean ± SD	Control group (*n* = 30), mean ± SD	Mean ± SE of difference	95% CI of difference (lower–upper)	*P*-value
CPB duration (min)	135.4 (SD: 28.51)	137.13 (SD: 22.3)	–1.73 ± 6.61	–14.96 to 11.49	0.794
ACC time (min)	117.97 (SD: 30.07)	119.77 (SD: 21.6)	–1.8 ± 6.76	–15.33 to 11.73	0.791
Ventilation time (h)	13.63 (SD: 3.64)	20.03 (SD: 6.77)	–6.4 ± 1.4	–9.21 to 3.59	**<0.001**
ICU stay (h)	39.3 (SD: 12.24)	50.93 (SD: 18.52)	–11.63 ± 4.05	–19.75 to –3.52	**0.006**
Hospital stay (days)	8.57 (SD: 1.77)	10.83 (SD: 3.27)	–2.27 ± 0.68	–3.63 to –0.91	**0.001**

ACC: aortic cross-clamp; CI: confidence interval; CPB: cardiopulmonary bypass; ICU: intensive care unit; SD: standard deviations; SE: standard error.

Bold/italic values represents statistically significant p value.

**Table 6:** Perioperative severity scores, blood loss and blood transfusion

Parameter	Haemoadsorption group (*n* = 20)	Control group (*n* = 20)	Mean ± SE of difference	95% CI of difference (lower–upper)	*P*-value
PF ratio					
T_1_	394.27 (SD: 80.23)	408.27 (SD: 60.01)	–14 ± 18.29	–50.62 to 22.62	0.447
T_2_	428.53 (SD: 61.89)	294.9 (SD: 51.02)	133.63 ± 14.61	104.32 to 162.95	**<0.001**
T_3_	392.97 (SD: 80.03)	278.73 (SD: 40.02)	114.23 ± 16.34	81.53 to 146.93	**<0.001**
T_4_	366.6 (SD: 68.52)	276 (SD: 35.73)	90.6 ± 14.11	62.36 to 118.84	**<0.001**
SOFA score					
T_1_	0.73 (SD: 1.2)	0.83 (SD: 1.23)	–0.1 ± 0.31	–0.73 to 0.53	0.752
T_2_	5.77 (SD: 1.83)	7.43 (SD: 1.43)	–1.67 ± 0.42	–2.52 to –0.82	**<0.001**
T_3_	2.57 (SD: 1.22)	3.27 (SD: 1.51)	–0.7 ± 0.35	–1.41 to 0.01	0.053
T_4_	0.67 (SD: 0.96)	1.27 (SD: 1.53)	–0.6 ± 0.33	–1.26 to 0.06	0.074
APACHE score					
T_1_	3.77 (SD: 2.11)	2.93 (SD: 2.00)	0.83 ± 0.53	–0.23 to 1.9	0.122
T_2_	10.63 (SD: 3.75)	10.9 (SD: 2.73)	–0.27 ± 0.85	–1.96 to 1.43	0.754
T_3_	4.37 (SD: 2.08)	4.37 (SD: .87)	0.0 ± 0.51	–1.02 to 1.02	1.000
T_4_	1.80 (SD: 1.54)	1.97 (SD: 1.45)	–0.17 ± 0.39	–0.94 to 0.61	0.668
S creatinine					
T_1_	1.05 (SD: 0.59)	1.13 (SD: 0.84)	–0.08 ± 0.19	–0.45 to 0.3	0.684
T_2_	1.15 (SD: 0.7)	1.18 (SD: 0.64)	–0.02 ± 0.17	–0.37 to 0.32	0.894
T_3_	1.09 (SD: 0.56)	1.21 (SD: 0.62)	–0.12 ± 0.15	–0.43 to 0.19	0.436
T_4_	1.00 (SD: 0.53)	1.14 (SD: 0.63)	–0.14 ± 0.15	–0.44 to 0.16	0.345
Blood loss intra-op	678.3 (SD: 257.8)	691.6 (SD: 207.6)	–13.33 ± 60.45	–134.3 to 107.6	0.826
Postoperative	228.3 (SD: 128.9)	258.5 (SD: 98.1)	–30.24 ± 33.38	–97.3 to 36.8	0.369
Blood transfusion					
RBC (units)	3.3 (SD: 1.97)	3.63 (SD: 1.79)	–0.33 ± 0.49	–1.31 to 0.64	0.495
FFP (units)	2.73 (SD: 1.05)	3.21 (SD: 1.4)	–0.47 ± 0.32	–1.12 to 0.17	0.146
SDP (units)	2.25 (SD: 0.46)	2.00 (SD: 0)	0.14 ± 0.37	–0.54 to 1.04	0.486

APACHE: acute physiology and chronic health evaluation; FFP: fresh frozen plasma; PF: partial pressure of arterial oxygen: fraction of inspired oxygen; RBC: red blood cells; SD: standard deviation; SDP: single-donor platelets; SE: standard error; SOFA: sequential organ failure assessment; T_1_: post-induction; T_2, 3, 4_: at 2, 24, 48 h after cardiopulmonary bypass.

Bold/italic values represents statistically significant p value.

**Table 7:** Comparison of haemodynamic parameters between the groups

Parameter	Haemoadsorption group (*n* = 30) Mean ± SD	Control group (*n* = 30) Mean ± SD	Mean ± SE of difference	95% CI of difference Lower Upper	*P*-value
MAP (mmHg)					
T_1_	81.63 (SD: 11.84)	85.07 (SD: 11.69)	–3.43 ± 3.04	–9.52 to 2.65	0.263
T_2_	79.20 (SD: 11.81)	78.50 (SD: 10.6)	0.7 ± 2.9	–5.1 to 6.5	0.810
T_3_	84.67 (SD: 10.39)	85.67 (10.65)	–1.0 ± 2.72	4.44 to –0.368	0.714
T_4_	85.37 (SD: 8.96)	86.97 (SD: 9.03)	–1.6 ± 2.32	3.05 to –0.689	0.493
CVP (mmHg)					
T_1_	9.07 (SD: 1.26)	8.43 (SD: 1.17)	0.63 ± 0.31	0.01 1.26	0.058
T_2_	9.7 (SD: 1.6)	10.6 (SD: 1.48)	–0.9 ± 0.4	–1.7 to –0.1	0.077
T_3_	9.77 (SD: 1.55)	10.37 (SD: 1.33)	–0.6 ± 0.37	–1.34 to 0.14	0.112
T_4_	9.3 (SD: 1.56)	10.03 (SD: 1.19)	–0.73 ± 0.36	–1.45 to –0.02	0.055
CO (L/min)					
T_1_	4.22 (SD: 1.52)	4.48 (SD: 1.38)	–0.25 ± 0.38	–1.01 to 0.50	0.502
T_2_	5.22 (SD: 1.18)	5.33 (SD: 1.48)	–0.11 ± 0.35	–0.81 to 0.59	0.752
T_3_	5.13 (SD: 1.2)	5.49 (SD: 1.18)	–0.35 ± 0.31	–0.97 to 0.27	0.259
CI (L/min/m_2_)					
T_1_	2.29 (SD: 0.73)	2.40 (SD: 0.66)	–0.11 ± 0.18	–0.47 to 0.26	0.553
T_2_	2.75 (SD: 0.65)	2.76 (SD: 0.74)	–0.01 ± 0.18	–0.37 to 0.36	0.969
T_3_	2.66 (SD: 0.5)	2.92 (SD: 0.72)	–0.25 ± 0.16	–0.58 to 0.07	0.123
SVR (dynes/s/cm^–5^)					
T_1_	1637 (SD: 567.7)	1348 (SD: 428.4)	288.7 ± 130.6	27.1 to 550.3	**0.031**
T_2_	1156 (SD: 347.2)	1113 (SD: 322.5)	43.5 ± 87.2	–131.1 to 218.1	0.620
T_3_	1190 (SD: 244.2)	1081 (SD: 247.2)	108.7 ± 64.0	–19.4 to 236.9	0.095

CI: cardiac index; CO: cardiac output; CVP: central venous pressure; MAP: mean arterial pressure; SD: standard deviation; SE: standard error; SVR: systemic vascular resistance; T1: post-induction; T_2, 3, 4_: at 2, 24, 48 h after cardiopulmonary bypass.

Bold/italic values represents statistically significant p value.

## DISCUSSION

In the current quasi-randomized study, we sought to evaluate the clinical effects of intraoperative HA during elective aortic root surgery. Intraoperative HA decreased the vasopressor need in the postoperative period as shown by a statistically significant reduction in the VIS. This translated into a reduction of the SOFA score in the immediate postoperative phase. Moreover, intraoperative HA was capable of mitigating the hyperinflammatory response leading to a significantly decreased IL-6 and PCT release. Finally, mechanical ventilation time, ICU and hospital stays were shorter in patients with intraoperative HA.

Contact of blood with artificial surfaces by use of CPB during cardiac surgery is associated with release of inflammatory cytokines leading to potentially adverse outcomes. Since inflammatory mediators are key triggers of inflammation and post-CPB hyperinflammation, the intra- or postoperative removal of such mediators from blood using blood purification with a cytokine adsorber has previously been described as a useful approach to control these hyperinflammatory processes [8, 9]. This approach has shown potential to prevent post-CPB systemic inflammatory response syndrome and multiple organ dysfunction syndrome. Longer CPB duration (>120 min) is expected in procedures such as aortic surgery, redo surgery or double-valve replacement with the potential risk of higher cytokine release. Conventional devices used during CPB are not capable of removing these cytokines. Hence, HA using the CytoSorb adsorber was developed for rapid elimination of cytokines.

In our pilot study evaluating 8 vs 8 patients, we could show that intraoperative HA significantly reduced IL-6 and PCT levels and also resulted in better clinical outcomes such a less inotropic support or ventilation time [5]. In the present study, we were able to confirm our initial pilot study results whereby we could show decreased levels of IL-6 and PCT with the use of intraoperative HA in patients undergoing major aortic surgery. This is an important finding as the results of the REmoval of Cytokines during CArdiac Surgery (RECCAS) study showed that IL-6 levels rise significantly after cardiac surgery and reach peak levels 2–4 h after termination of CPB [10]. This was proven by the present analysis, as we found peak IL-6 levels at the T_2_ timepoint, i.e. 2 h after termination of CPB. In addition to this, in a large cohort of patients undergoing elective myocardial revascularization, Deppe *et al.* showed reduced cytokine (IL-6) burden and attenuated inflammatory response in HA-treated patients [11].

Elevated levels of IL-6 correlate well with postoperative inflammatory response, myocardial ischaemia, low CO and requirement for vasopressors [2, 12]. Although, the MAP and CO values did not show any differences in our study, the requirement for noradrenaline and the VIS to maintain adequate organ perfusion was higher in the control group compared to the HA group. In a retrospective propensity score-matched analysis by Saller *et al.*, intraoperative HA significantly reduced the need for noradrenaline (HA: 0.102 µg/kg/min, C: 0.113 µg/kg/min, *P* = 0.043), the amount of blood transfusions, and improved acid–base balance in aortic surgery with hypothermic circulatory arrest [13]. This is in line with the results of the present analysis where significantly reduced noradrenaline requirements (HA: 0.03 µg/kg/min vs C: 0.08 µg/kg/min, *P* = 0.004 at T_2_, and HA: 0.02 µg/kg/min vs C: 0.04 µg/kg/min, *P* = 0.004 at T_3_) were observed. These significant reductions in inotropic support translated into a significant reduction of the VIS in the HA-treated group. Few studies reported better stabilization of haemodynamic parameters, both intraoperatively and postoperatively, as demonstrated by reduced catecholamine support (adrenaline and noradrenaline) and an increase in MAP [14, 15]. In a retrospective case series by Calabrò *et al.*, involving 40 cardiac surgery ICU patients with multiple organ dysfunction syndrome mainly due to cardiogenic shock (28/40), use of CytoSorb resulted in 30-day mortality of 55% and ICU mortality of 52.5%, compared to an expected ICU mortality of 80% [16]. There was significant reduction of the VIS after 48 h of treatment as compared to baseline (20 vs 10, *P* = 0.009). In a recent, observational study of patients undergoing complex cardiac surgery, Manohar *et al.* showed lower VIS in the HA group (HA: 3.5 vs C: 5.5, *P* = 0.05) compared to controls [17].

Furthermore, from a clinical perspective, we could show better organ function and faster recovery with the use of HA in terms of better PF ratio, decreased duration of mechanical ventilation, shorter ICU and hospital length of stay compared to the control group. Despite a large number of patients, Saller *et al.* did not have enough data on IL-6 levels, nor did they find any differences in length of hospital stay or survival in their study [13]. Hence, the present analysis is the 1st to prospectively evaluate IL-6 and PCT levels comprehensively in major aortic surgery. In a recently published randomized controlled trial, Doukas *et al.* reported a shorter ventilation time in the HA group (HA: 80 h vs C: 510 h, *P* = 0.08) and a significantly less need for prolonged ventilation time compared to controls (1 HA group vs 9 C group, *P* = 0.03) in patients undergoing thoraco-abdominal aneurysm repair [18]. It would be interesting to study extravascular lung water and total fluid balance in each group of patients in forthcoming studies, as these are among the major factors affecting postoperative pulmonary function.

In a recently published systematic review and meta-analysis, Becker *et al.* found no evidence for a positive effect of the CytoSorb adsorber on mortality across a variety of diagnoses that justifies its widespread use in intensive care medicine [19]. In fact, they found significant survival advantage in untreated controls in patients with cardiac arrest. Moreover, they found no significant differences in the ICU stay, hospital stay, IL-6 levels, PCT, c-reactive protein, lactate level, MAP and noradrenaline dosage between the HA group and control group. Another meta-analysis published by Heymann *et al.* concluded that the use of CytoSorb HA does not exhibit a consistent decrease in IL-6 and other inflammatory parameters within the 1st 5 days of treatment [20]. Many other studies have shown no evidence of reduction in proinflammatory cytokines or clinical benefits in patients after cardiac surgery, including an randomized controlled trial conducted by Asch *et al.* in patients with infective endocarditis [21]. Our major concerns for these studies include a small sample size, which could have adversely affected statistical results (*n* = 20 with 10 patients in HA group and 10 in control group in Asch *et al.*’s study), heterogeneity of the population studied (analysis of patients with sepsis, CPB surgery, SARS-COV-2 and cardiac arrest), inclusion of older studies and exclusion of recent trial by Jansen *et al.* in the analysis. A highly variable effect of HA therapy in these studies can be explained by the fact that the cytokine activation of CPB is associated with substantial inter-individual differences. Second, depending on plasma concentration, cytokine plasma levels are reduced less effectively when peak concentrations are low. The study by Jansen *et al.* is probably the most relevant study in regard to impact of CytoSorb on cytokine levels [3]. In a randomized study, they have demonstrated marked attenuation of plasma cytokines in a highly reproducible model of pronounced systemic inflammation. To state that HA therapy does not benefit aortic aneurysm patients may not be true as the results of present study, our previous pilot study, and a much larger study by Saller *et al.* have shown beneficial outcome of this therapy [5, 13]. Furthermore, a homogeneous population in the present study possibly had a beneficial influence in terms of confounding factors.

The primary limitation of this study is that it was not a real randomized trial, and it included data only from a single centre. We did not evaluate the effect of the HA device on coagulation parameters. In addition, we included patients with aortic aneurysms presenting for elective aortic root surgery. It would be worthwhile to include subjects with acute aortic dissection undergoing emergency surgery, as they generally manifest with a more severe inflammatory response. Finally, we tried to exclude confounders published in previous studies, as unknown confounders might have biased our results. Nevertheless, our data may help those who are planning to design prospective, randomized trials in this patient population.

## CONCLUSION

In this prospective, observational study, we used the HA (CytoSorb) device as an adjunctive therapy for addressing the excessive cytokines in patients undergoing aortic surgery. We observed that HA therapy was associated with improved haemodynamics as shown by the decreased VIS and levels of IL-6, better PF ratio, reduced duration of mechanical ventilation, shorter ICU stay and shorter hospital stay compared to controls. However, these results must be evaluated in larger cohorts. These positive results may provide valuable help for defining the rationale of end-points for future randomized trials in this patient population.

## Data Availability

All relevant data are contained within the manuscript.

## Notes

Presented at the 37th Annual Meeting of the European Association for Cardio-Thoracic Surgery, Vienna, Austria, 4–7 October 2023.

## ABBREVIATIONS

CO: Cardiac output
CPB: Cardiopulmonary bypass
HA: Haemoadsorption
ICU: Intensive care unit
IL: Interleukin
MAP: Mean arterial pressure
PCT: Procalcitonin
PF ratio: Partial pressure of arterial oxygen/fraction of inspired oxygen ratio
RECCAS: REmoval of Cytokines during CArdiac Surgery
SOFA: Sequential organ failure assessment
T_1_: Baseline time point, post-induction of anaesthesia
T_2, 3, 4_: Time point at 2, 24, 48 h after discontinuation of CPB
